# Estrogen ameliorates microglial activation by inhibiting the Kir2.1 inward-rectifier K^+^ channel

**DOI:** 10.1038/srep22864

**Published:** 2016-03-10

**Authors:** Shih-Ying Wu, Yun-Wen Chen, Sheng-Feng Tsai, Sheng-Nan Wu, Yao-Hsiang Shih, Ya-Fen Jiang-Shieh, Ting-Ting Yang, Yu-Min Kuo

**Affiliations:** 1 Institute of Basic Medical Sciences, National Cheng Kung University, Tainan, Taiwan; 2 Department of Cell Biology and Anatomy, National Cheng Kung University, Tainan, Taiwan; 3 Department of Chinese Medicine for Post-Baccalaureate, I-Shou University, Kaohsiung, Taiwan; 4 Department of Physiology, National Cheng Kung University, Tainan, Taiwan

## Abstract

Microglial activation is implicated in the pathogenesis of Parkinson’s disease (PD). Although the etiology of PD remains unclear, age and male gender are known PD risk factors. By comparing microglia and dopaminergic (DA) neurons in the substantia nigra (SN) of male and female mice of different ages, we found that the degrees of microglial activation and DA neuron loss increased with age in both genders, but were more pronounced in males, as were peripheral lipopolysaccharide (LPS)-induced microglial activation and DA neuron loss. A bilateral ovariectomy (OVX) eliminated the female-associated protection against age- and LPS-induced microglial activation, which suggests that ovary hormones are involved in gender-specific responses. Treating female mice with 17β-estradiol supplements reduced the age-associated microglial activation in OVX mice. Moreover, pretreating mouse BV2 microglial cells with 17β-estradiol inhibited LPS-induced elevation of Toll-like receptor 4, phosphorylated p38, and TNF-α levels. We then examined the effect of 17β-estradiol on inward-rectifier K^+^ channel Kir2.1, a known regulator of microglial activation. We found that 17β-estradiol inhibited the Kir2.1 activity of BV2 cells by reducing the probability that the channel would be open. We conclude that age- and inflammation-associated microglial activation is attenuated by ovarian estrogen, because it inhibits Kir2.1.

Parkinson’s disease (PD) is an age-related neurodegenerative disease. Pathologically, PD is characterized by a selective loss of dopaminergic (DA) neurons in the substantia nigra (SN) pars compacta^1^,^2^. Epidemiological studies indicate that about 5% of PD cases occur in familial clusters with early-onset symptoms, while the majority of PD cases are sporadic with a late-onset age between 50 and 60 years old^1^,^3^. Although, the exact etiology for late-onset sporadic PD is not clear, recent studies^2^,^4^ have associated the pathogenesis of PD with microglial activation. Activated microglia in the SN and striatum have been shown in the post-mortem pathology examinations of the brains of patients with PD^4^,^5^ and in positron emission tomography (PET) images of living patients with PD^6^. The degree of midbrain microglial activation is negatively correlated with the level of dopamine transporter in the striatum and positively correlated with the motor severity in the early stage of PD^6^. In addition, microglia are activated and DA neurons are lost in the SN in animal models of PD, including those that require the direct administration of the immunogen lipopolysaccharide (LPS) into the brain^7^,^8^. Intranigral infusion of LPS induces microglial activation and then leads to DA neuron degeneration and death^7^,^8^,^9^. Using anti-inflammatory drugs to inhibit LPS-induced microglial activation and related inflammatory responses reduces the injury to DA neurons^10^. More important, epidemiological studies have reported that the incidence of idiopathic PD is much lower in chronic users of nonsteroidal anti-inflammatory drugs than in age-matched nonusers^11^,^12^. These findings suggest that microglial activation contributes to DA neuron death.

Another important risk factor for late-onset sporadic PD is gender. Both the incidence and the prevalence of PD are 1.5–2 times higher in men than in women^13^,^14^. The average age of onset in women is several years later than in men^13^. The differential gender effect has been attributed to the female sex hormones, especially estrogen^15^. Estrogen, a potent neurotrophic agent, is known to induce an anti-apoptosis reaction, promote neuron survival, and increase both neurotransmission synthesis and synaptic connections^15^,^16^,^17^,^18^. However, compared with the rich information of estrogen-induced neuronal responses, the physiological function of estrogen in other brain cells, such as microglia, is less clear.

Under physiological conditions, microglia exist in a resting state characterized by ramified morphology^19^,^20^. However, the term “resting microglia” is misleading because resting microglia are always dynamic^19^,^20^,^21^. After they have been exposed to certain stimulatory signals, microglia activate in order to execute innate immune functions^20^,^21^,^22^. This includes changes in morphology, gene expression, and functional behavior^21^,^22^. In contrast, numerous anti-inflammatory agents are also known to inhibit microglial activation or to return already active microglia to their resting state^22^,^23^,^24^. It has been shown that microglia express estrogen receptors^25^,^26^, and that estrogen or estrogen receptor ligands significantly inhibit the production of LPS-induced proinflammatory cytokines and the proliferation and activation of microglia in culture^26^,^27^. Thus, the estrogen-associated regulation of microglia might also participate in the estrogen-induced neuroprotective effect, especially in DA neurons, which are relatively sensitive to inflammation-induced injury^28^.

Exactly how estrogen regulates the activation of microglia is still unclear. Recently, ion channel activities, especially inward-rectifier K^+^ channel Kir2.1, have been shown to affect microglial activation^24^,^29^. Kir2.1, which is constitutively expressed in microglia and macrophages, helps to maintain a negative membrane potential, which regulates the influx of Ca^2+^ and subsequent signaling pathways associated with microglial activation^24^,^30^. We investigated the effect of estrogen on age-associated microglial activation. The role of Kir2.1 in the estrogen-induced regulation of microglia was also examined. Initially, we characterized the changes of microglial activation in the SN of 3-, 6-, 9-, and 12-month-old male and female mice because activated microglia were already evident in 4-month-old male C57BL/6 mice^31^. To examine the effects of estrogen on the degree of microglial activation, female mice were bilaterally ovariectomized and then given 17β-estradiol (E2) supplements. The effect of E2 on microglial activation was also investigated in a BV2 mouse microglial cell line.

## Results

### Effects of gender on age-related microglial activation and DA neuron loss in the SN

**Figure 1:**
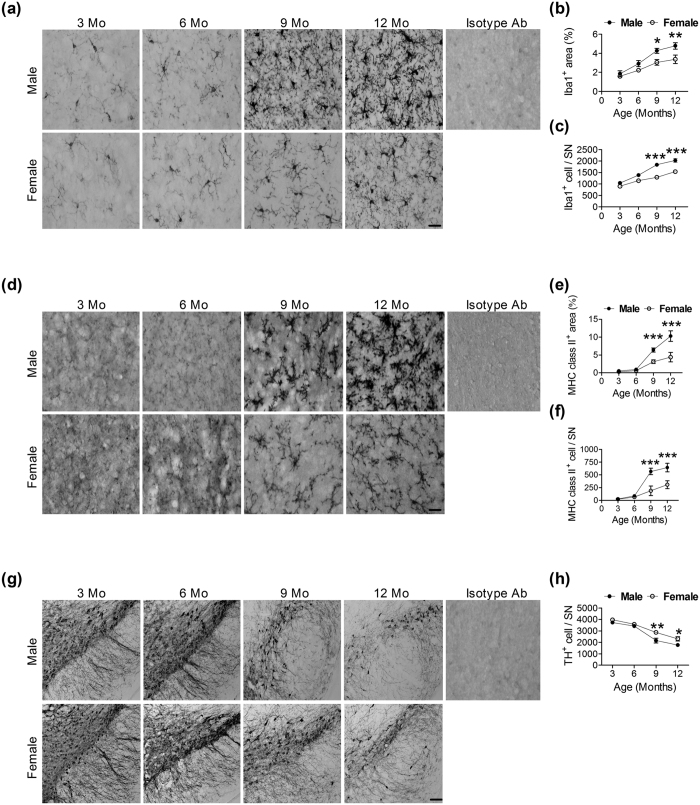
Temporal profiles of microglia and DA neurons in the SN of female and male C57BL/6J mice. (**a**) Representative immunostained micrographs show Iba1^+^ cells in the SN of 3-, 6-, 9-, and 12-month-old female and male mice. Scale bar: 10 μm. Quantitative results of Iba1^+^ cell areas (**b**) and the number of Iba1^+^ cells (**c**) in the SN of female and male mice at different ages (n = 5). *(*p* < 0.05), **(*p* < 0.01), ***(*p* < 0.001): versus the opposite sex. (**d**) Representative immunostained micrographs show MHC class II^+^ cells in the SN of 3-, 6-, 9-, and 12-month-old female and male mice. Scale bar: 10 μm. Quantitative results of MHC class II^+^ cell areas (**e**) and the number of MHC class II^+^ cells (**f**) in the SN of female and male mice at different ages (n = 5). ***(*p* < 0.001): versus the opposite sex. (**g**) Representative immunostained micrographs show TH^+^ cells in the SN of 3-, 6-, 9-, and 12-month-old female and male mice. Scale bar: 200 μm. (**h**) Quantitative results of TH^+^ cells in the SN of female and male mice at different ages (n = 5). *(*p* < 0.05), **(*p* < 0.01): versus the opposite sex.

To establish the temporal profiles of microglial activation and DA neuron loss in the SN during aging, we harvested the brains of 3-, 6-, 9- and 12-month-old male and female C57BL/6 J mice. The intensity of the Iba1^+^ signal increased with age in both genders (Fig. 1a). The Iba1^+^ cells in the SN of 12-month-old mice had intensified Iba1^+^ signals in the cell bodies and processes. Quantitative analysis showed that age (*F* = 26.5, *df* 3/32, *p* < 0.001) and gender (*F* = 18.7, *df* 1/32, *p* < 0.001) both affected the total Iba1^+^ cell area, but that there was no significant interaction between them (*F* = 1.6, *df* 3/32, *p* = 0.209) (Fig. 1b). Post-hoc tests revealed that 9- and 12-month-old male mice had larger Iba1^+^ cell areas than did female mice (Fig. 1b). In addition to morphological changes, the older the mice were, the more Iba1^+^ cells they had in the SN, regardless of gender (*F* = 76.6, *df* 3/32, *p* < 0.001); the gender effect was significant (*F* = 76, *df* 1/32, *p* < 0.001) (Fig. 1c). Confocal micrographs revealed that Iba1^+^ cells in the SNs of 9-month-old mice had more (hyper-ramified) processes than did those of 6-month-old mice (Fig. S1).

The age-related change in microglial status was also evaluated using CD11b, another microglia marker. CD11b^+^ cell immunoreactivities increased with age in both genders (Fig. S2a). The CD11b^+^ areas were very small before the mice were 6 months old, but they significantly increased after the mice turned 9 months old (Fig. S2b). A significant gender effect (*F* = 51, *df* 1/32, *p* < 0.001) was also evident (Fig. S2c), and the number of CD11b^+^ cells significantly rose after the mice turned 9 months old (Fig. S2c).

We also used major histocompatibility complex (MHC) class II, a marker for activated microglia, to confirm the effect of age-related microglial activation in the SN (Fig. 1d). The interactions between age and gender on the area (*F* = 30.7, *df* = 3/32, *p* < 0.001; Fig. 1e) and the number (*F* = 29.9, *df* 3/32, *p* < 0.001; Fig. 1f) of MHC class II^+^ cells were significant. Bonferroni post-hoc analysis revealed that 9- and 12-month-old male mice had larger MHC class II^+^ areas and more MHC class II^+^ cells than did female mice.

For DA neurons, TH^+^ cell immunoreactivities were observed in the cell bodies and neurites of the SN (Fig. 1g). Two-way ANOVA showed that both age (*F* = 76.3, *df* 3/32, *p* < 0.001) and gender (*F* = 18.3, *df* 1/32, *p* < 0.001) affected the number of TH^+^ cells, but that there was no significant interaction between age and gender (*F* = 1.8, *df* 3/32, *p* = 0.171) (Fig. 1h). Bonferroni post-hoc tests indicated that the male mice had fewer TH^+^ cells than did the female mice at 9 and 12 months old (Fig. 1h).We also determined the temporal profiles of microglial activation and DA neuron loss in 3-, 6-, and 9-month-old male and female BALB/c mice. The interaction between age and gender on the area (*F* = 12.8, *df 2*/18, *p* < 0.001; Fig. S3a) and the number (*F* = 9.8, *df* 2/18, *p* = 0.001; Fig. S3b) of Iba1^+^ cells and the number of TH^+^ cells (*F* = 5.7, *df* 2/18, *p* = 0.012; Fig. S3c) in the SN of BALB/c mice were significant. Post-hoc tests indicated that the male mice had larger and more Iba1^+^ cells, but fewer TH^+^ cells than did the female mice at 9 months old. These results suggest that the age-related microglia activation and DA neuron loss have similar patterns across different inbred strains of mice.

### Effects of gender on inflammation-induced microglial activation and DA neuron loss in the SN of young mice

**Figure 2:**
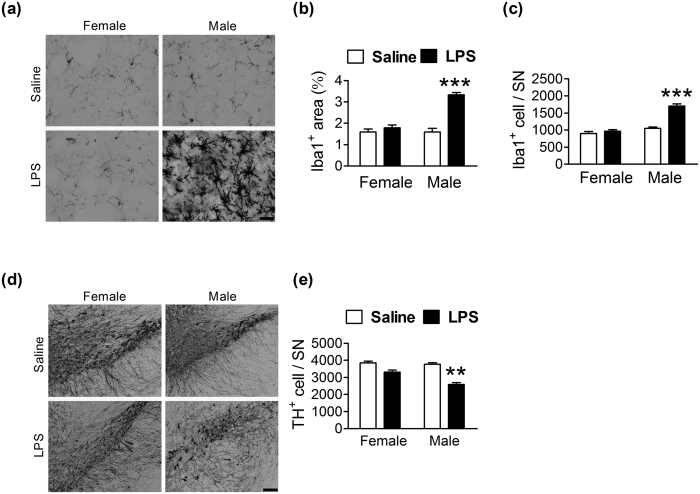
Differential effects of LPS on microglial activation and DA neuron loss in the SN of female and male mice. (**a,d**) Representative immunostained micrographs show Iba1^+^ (**a**) and TH^+^ (**d**) cells in the SN of female and male mice 1 day after an LPS (0.15 mg/kg of body weight, i.p.) injection. Scale bar: 10 μm in (**a**); 200 μm in (**d**). Quantitative results of Iba1^+^ cell areas (**b**), the number of Iba1^+^ cells (**c**) and the number of TH^+^ cells (**e**) in the SN of female and male mice 1 day after an LPS injection (n = 5–6). **(*p* < 0.01), ***(*p* < 0.001): versus the respective Saline control.

We next compared the inflammatory responses of mice of both genders to the same stimulus (a peripheral LPS injection). Initially, 3-month-old male mice were treated with different concentrations of LPS (0, 0.05, 0.1, 0.15 mg/kg, i.p.). Twenty-four hours later, only those mice that had been given the highest dose of LPS (0.15 mg/kg) showed increases in area (*F* = 14.7, *df* 3/15, *p* < 0.001; Fig. S4a) and the number (*F* = 31.4, *df* 3/15, *p* < 0.001; Fig. S4b) of Iba1^+^ cells and reductions in the number of TH^+^ cells (*F* = 10.0, *df* 3/15, *p* = 0.001; Fig. S4c) in the SN. We then used the same protocol (0.15 mg/kg of LPS, i.p., 24 h post-injection interval) to challenge a different set of male and female mice. The interactions between LPS and gender on the area (*F* = 30.6, *df* = 1/17, *p* < 0.001; Fig. 2a,b) and the number (*F* = 32.7, *df* 1/17, *p* < 0.001; Fig. 2a,c) of Iba1^+^ cells were significant. Bonferroni post-hoc analysis showed that the area (Fig. 2b) and number (Fig. 2c) of Iba1^+^ cells were larger after LPS treatment, but only in male mice. Likewise, interactions between LPS and gender on the area (*F* = 34.8, *df* = 1/17, *p* < 0.001; Fig. S5a,b) and number (*F* = 22.7, *df* 1/17, *p* < 0.001; Fig. S5a,c) of CD11b^+^ cells were significant. Post-hoc analysis showed that only male mice had larger areas (Fig. S5b) and a greater number (Fig. S5c) of CD11b^+^ cells after LPS treatment. Along with the responses of microglia, LPS-induced reduction of TH^+^ cells occurred only in the SN of male mice (Figs 2d,e).

### Effects of sex hormones on female-related protection against LPS-induced microglial activation and DA neuron loss in the SN

**Figure 3:**
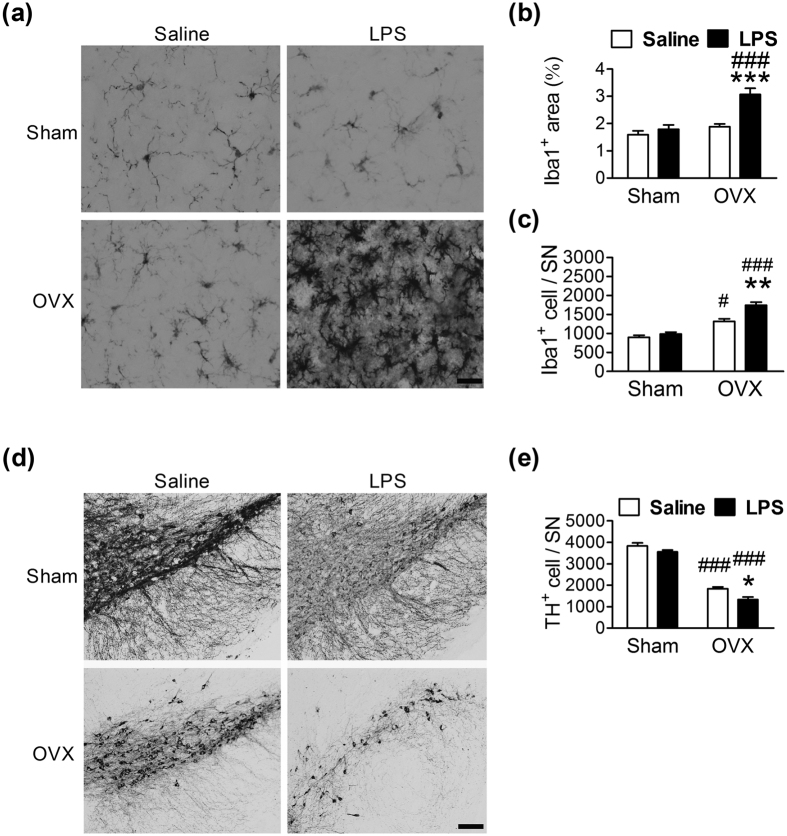
Effects of ovaries on LPS-induced microglial activation and DA neuron loss in the SN of 3-month-old female mice. (**a,d**) Representative immunostained micrographs show Iba1^+^ (**a**) and TH^+^ (**d**) cells in the SN of female mice 1 day after an LPS (0.15 mg/kg of body weight, i.p.) injection. OVX: bilateral ovariectomy. Scale bar: 10 μm in (**a**); 200 μm in (**d**). Quantitative results of Iba1^+^ cell areas (**b**), the number of Iba1^+^ cells (**c**) and the number of TH^+^ cells (**e**) in the SN of female mice 1 day after an LPS injection (n = 5). *(*p* < 0.05), **(*p* < 0.01), ***(*p* < 0.001): versus respective Saline group. ^#^(*p* < 0.05), ^###^(*p* < 0.001): versus respective Sham group.

We hypothesized that sex hormones contribute to the differential responses of microglia in males and females under inflammatory stimulation. To investigate the effects of ovarian hormones, 2-month-old female mice underwent a bilateral ovariectomy (OVX). One month after the surgery, the mice were given a single LPS injection (0.15 mg/kg, i.p.) and were killed 24 h later. LPS did not affect the general morphology of microglia in the Sham mice, while OVX increased LPS-induced microglial activation (Fig. 3a). Quantitative analysis showed that LPS had significantly increased the area (*F* = 23.4, *df* 1/16, *p* < 0.001; Fig. 3b) and the number (*F* = 86.3, *df* 1/16, *p* < 0.001; Fig. 3c) of Iba1^+^ cells after an OVX. Furthermore, the OVX exacerbated the LPS-induced reduction of the number of TH^+^ cells (Fig. 3d,e). Post-hoc analysis indicated that OVX treatment alone increased the number of Iba1^+^ cells (Fig. 3c, open bar) and decreased the number of TH^+^ cells (Fig. 3e, open bar) in the SN.

### Effects of estrogen on age-related and LPS-induced microglial activation and DA neuron loss in the SN

**Figure 4:**
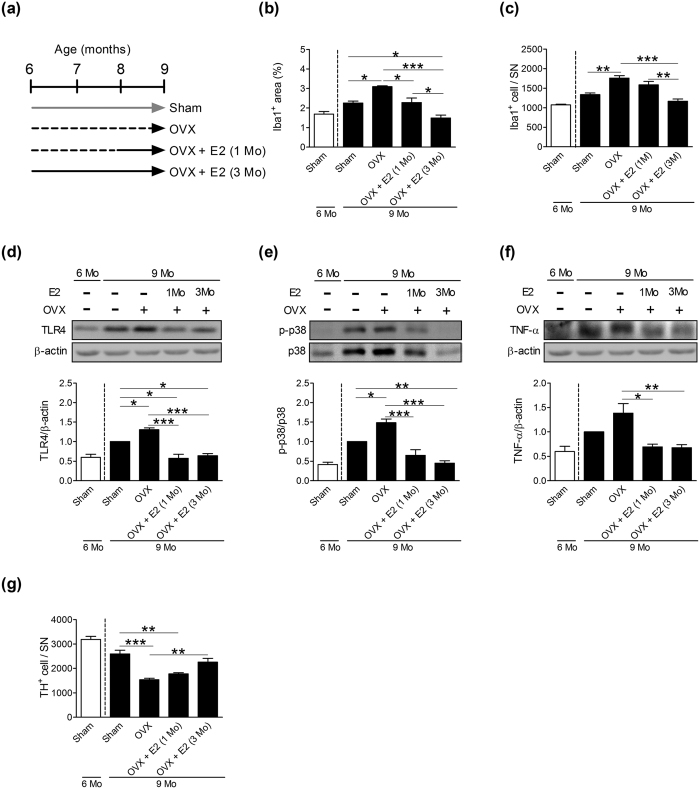
Effects of 17β-estradiol (E2) on ovariectomy-induced microglial activation in the SN of 9-month-old female mice. (**a**) Scheme of the experimental design. Six-month-old female mice were given a bilateral ovariectomy (OVX). Mice given the same surgical incisions but without removing the ovaries were Sham controls. OVX + E2 (1 Mo): OVX mice treated with E2 supplements for 1 month when they turned 8 months old. OVX + E2 (3 Mo): OVX mice treated with E2 supplements for 3 months immediately after the OVX. Solid line: OVX with E2 supplements. Quantitative results of Iba1^+^ area (**b**), the number of Iba1^+^ cells (**c**), levels of TLR4 (**d**), levels of p-p38 (**e**), levels of TNF-α (**f**) and the number of TH^+^ cells (**g**) in the SN of female mice. The open bar represents 6-month-old Sham mice, while the other solid bars are from 9-month-old mice (n = 5). *(*p* < 0.05), **(*p* < 0.01), ***(*p* < 0.001).

Based on the observations that the degree of microglial activation and the number of DA neurons were similar between 6-month-old male and female mice, but were different in 9-month-old mice (Fig. 1), we then gave 6-month-old female mice an OVX and treated them with 17β-estradiol (E2) supplements for the next 3 months [OVX + E2 (3Mo), Fig. 4a]. The area (Fig. 4b) and number (Fig. 4c) of Iba1^+^ cells in 9-month-old OVX mice were larger than those in 9-month-old Sham controls. Moreover, levels of innate immune-related molecule Toll-like receptor (TLR) 4 (Fig. 4d) and MAP kinase p38 (Fig. 4e), but not TNF-α (Fig. 4f) were increased in 9-month-old OVX mice. An ovariectomy also reduced the number of TH^+^ cells (Fig. 4g). Three months of E2 supplements completely antagonized the ovariectomy-induced changes in microglia (Fig. 4b,c), TLR4 (Fig. 4d), p38 (Fig. 4e), TNF-α (Fig. 4f), and DA neurons (Fig. 4g).

To mimic E2 supplementation after menopause, some OVX mice were left without any treatment for 2 months until they turned 8 months old, and then they were given E2 supplements for 1 month [OVX + E2 (1Mo), Fig. 4a]. OVX + E2 (1Mo) mice had smaller Iba1^+^ cell area (Fig. 4b), lower levels of TLR4 (Fig. 4d), p38 (Fig. 4e) and TNF-α (Fig. 4f) than did the OVX mice, but the number of Iba1^+^ (Fig. 4c) and TH^+^ (Fig. 4g) cells did not change by 1-month of E2 supplements.

**Figure 5:**
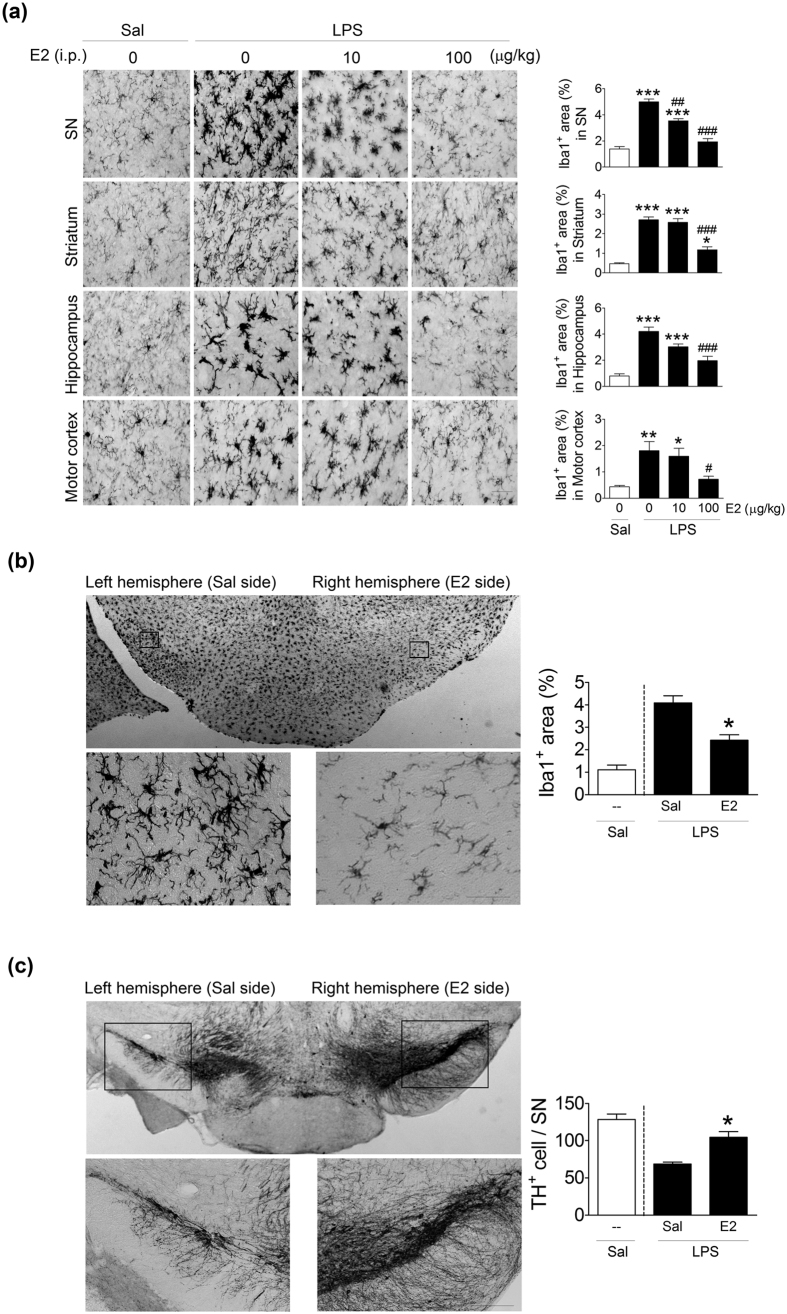
Effects of 17β-estradiol (E2) on LPS-induced microglial activation and DA neuron loss in various brain regions of male mice. (**a**) Representative immunostained micrographs show Iba1^+^ cells in the SN, striatum, hippocampus and motor cortex of 3-month-old mice 1 day after an LPS (0.15 mg/kg of body weight, i.p.) or saline (Sal) injection. One hour before the LPS injection, mice were injected with 0, 10 or 100 μg/kg body weight of E2. Quantitative data are shown in the respective right panels. Scale bar: 20 μm. (n = 4). *(*p* < 0.05), **(*p* < 0.01), ***(*p* < 0.001): versus the Sal group; ^#^(*p* < 0.05), ^##^(*p* < 0.01), ^###^(*p* < 0.001): versus the LPS group. (**b,c**) Changes in Iba1^+^ and TH^+^ cells in the SN of 3-month-old male mice 1 days after an intraperitoneal (i.p.) LPS injection. One hour before the LPS injection, the right SN was injected with E2, and the left with an equal amount of Sal. Representative micrographs are shown in the upper panels. The lower panels are enlargements of the boxes in the upper panels. Quantitative data for a fixed region (0.049 mm^2^) immediately next to the injection site are shown in the right panels (n = 4). *(*p* < 0.05): versus the Sal injection side of LPS treatment group. Sal(i.p.): a group of 3-month-old mice given an intraperitoneal injection of saline but no intra-SN injection. The Sal(i.p.) group was a reference group and was not included in the statistical analysis.

To further characterize the effect of E2 on microglia *in vivo*, 3-month-old male mice were given intraperitoneal (i.p.) injections of 0, 10, or 100 μg/kg of E2 1 hour before they were injected with LPS (0.15 mg/kg, i.p.). The LPS-induced increases in Iba1^+^ area in four brain regions, including the SN, striatum, hippocampus, and motor cortex, were significantly attenuated in E2-injected mice (Fig. 5a). Post-hoc analysis indicated that 100 μg/kg of E2 effectively suppressed the LPS-induced increases in Iba1^+^ area in these four brain regions.

In another set of of experiments, 1 hour before 3-month-old male mice were injected (i.p.) with LPS, their right SNs were perfused with E2, while their left SNs were injected with an equal volume of saline. The LPS-induced increases in area of Iba1^+^ cells were significantly attenuated (Fig. 5b) and the LPS-induced losses of TH^+^ cells were significantly downregulated (Fig. 5c) on the E2-injected side.

### Effects of estrogen on LPS-induced inflammation in BV2 microglial cells

**Figure 6:**
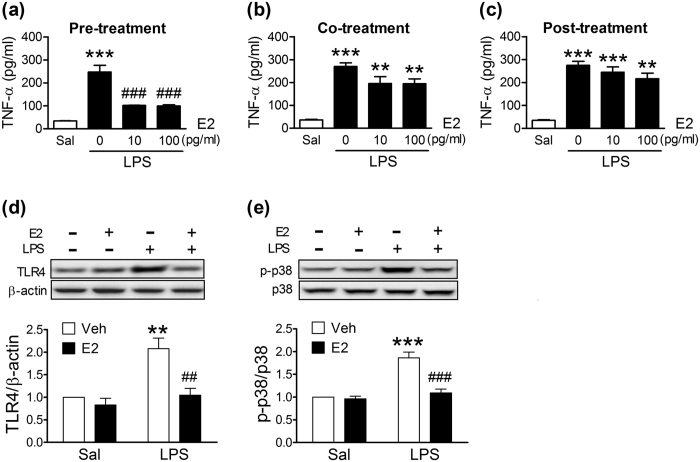
Effects of 17β-estradiol (E2) on LPS-induced inflammation in the BV2 microglial cells. The production of TNF-α in the BV2 cells given E2 30 min before (**a**, Pre-treatment), together (**b**, Co-treatment), and 30 min after (**c**, Post-treatment) the LPS treatment (n = 4). **(*p* < 0.01), ***(*p* < 0.001): versus respective Saline controls. ^###^(*p* < 0.001): versus no E2 treatment (0) group. Effects of E2 pre-treatment on LPS-induced elevations of TLR4 (**d**) and p-p38 (**e**). Representative immunoblots are shown in the upper panels, and quantitative data in the respective lower panels. (n = 3). **(*p* < 0.01), ***(*p* < 0.001): versus respective Saline group. ^##^(*p* < 0.01), ^###^(*p* < 0.001): versus respective Vehicle (Veh) control group.

To clarify the role of estrogen in microglial activation, BV2 cells, a microglial cell line, were treated with various doses of E2 before (pre-treatment), together (co-treatment), and after (post-treatment) adding LPS (10 ng/ml) to the culture media. The LPS-induced production of TNF-α was inhibited in E2-pretreated BV2 cells (Fig. 6a), but not in co-treated (Fig. 6b) or post-treated (Fig. 6c) (up to 100 pg/ml) BV2 cells. LPS-induced upregulation of TLR4 (Fig. 6d) and p-p38 (Fig. 6e) were also inhibited in E2-pretreated BV2 cells.

### Effects of E2 on Kir2.1 channel activity in BV2 microglial cells

**Figure 7:**
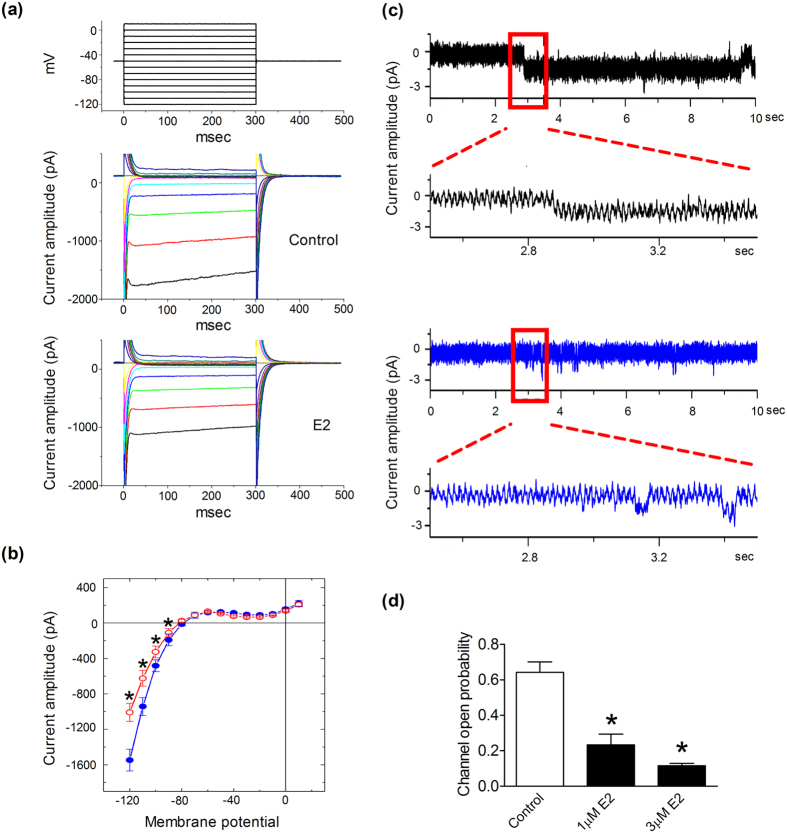
Inhibitory effect of 17β-estradiol (E2) on the activity of inwardly rectifying K^+^ channel 2.1 recorded from BV2 microglia. (**a**) In this set of experiments, the cells were held at −50 mV, and various potentials ranging from −120 to +10 mV in 10-mV increments were applied (upper panel). Original current traces obtained from cells in the absence (Control) and presence of E2. (**b**) Average current-voltage (*I-V*) relations of I_K(IR)_ in the absence (blue circles) and presence (red circles) of E2 (n = 5–9). * (*p* < 0.05): versus controls measured at the same level of voltage. Adding E2 significantly suppressed the I_K(IR)_ amplitude measured at the voltage ranging between −90 and −120 mV. (**c**) Original single-channel currents obtained in the absence (**black trace**) and presence (**blue trace**) of 3 μM of E2. The attached cell was held at 0 mV relative to the bath. Channel opening gives a downward deflection in current. The boxed regions are enlarged and shown beneath the original trace. (**d**) Summary of the data showing the dose-dependent effect of E2 on the probability of the Kir channel opening (n = 9–10). * (*p* < 0.05): versus Controls measured at the same level of voltage. Each cell was held at 0 mV.

Because the Kir2.1 channel is known to control microglial membrane potential and mediate a variety of signals associated with microglial activation^24^, we then examined the effect of E2 on inwardly rectifying K^+^ current (I_K(IR)_) in BV2 cells. The amplitude of I_K(IR)_ in E2-treated (3 μM) BV2 cells was lower (Fig. 7a) and significantly lower between −90 mV and −120 mV (Fig. 7b). To determine the mechanism, BV2 cells were bathed in Ca^2+^ -free Tyrode’s solution and a cell-attached configuration was made. The activity of Kir2.1 channels (changes in single-channel amplitude) could be readily observed (Fig. 7c, black trace). When E2 was included in the recording pipette, the probability of Kir2.1 channel openings was significantly lower than normal (Fig. 7c, blue trace). The inhibitory effect of E2 on the probability of Kir2.1 channel openings was dose-dependent (Fig. 7d).

## Discussion

After examining the effects of gender on age- and inflammation-induced microglial activation and DA neuron loss in the SN of mice, we found that they were higher with age in both genders and more pronounced in males, as were peripheral LPS-induced microglial activation and subsequent DA neuron loss. The female-related protections against age- and LPS-associated injuries were eliminated by an OVX, and E2 supplements reversed OVX-induced microglial activation and DA neuron loss in female mice. Furthermore, pretreatment with E2 significantly inhibited LPS-induced microglial activation *in vivo* and *in vitro*. The mechanism through which E2 inhibits microglial activation is partially due to its ability to prevent the Kir2.1 channel from opening. Taking these findings together indicate that estrogen contributes to age- and inflammation-associated microglial activation and subsequently to the survival of DA neurons.

Consistent with other reports^32^,^33^, we showed that microglial activation, as indicated by higher expression levels of Iba1, CD11b and MHC class II and by greater number of Iba1^+^, CD11b^+^ and MHC class II^+^ cells, was age-dependent. Age-associated microglial activation might be induced by abnormal protein aggregates (e.g., α-synuclein)^34^, a dysfunction of CD200-CD200R-mediated microglial silencing^25^, increased peripheral inflammation^35^, or a combination of these. We found that age-associated increases of microglial activation were less pronounced in female mice, which suggested that their sex hormones are involved in microglial activation. We hypothesized that E2 antagonizes age-associated microglial activation, which was supported by the results of our treatment of OVX mice with E2 supplements.

The anti-inflammatory effect of estrogen has been reported. In several animal models of neurological disease, such as multiple sclerosis, spinal cord injury, and experimental autoimmune encephalomyelitis, estrogen supplements or synthetic estrogen receptor ligands reduced the severity of neuronal injury^36^,^37^,^38^. Although the causal relationship awaits clarification, the authors of these 4 cited studies attributed some of the protective effect of estrogen in these reports to anti-inflammation, because microglial activation and proinflammatory cytokine levels were downregulated^26^,^37^,^38^,^39^. Our results also showed that peripheral and central E2 treatments effectively inhibited LPS-induced microglial activation and DA neuron loss. Using a microglial cell line culture, we studied the mechanism for the anti-inflammatory effect of estrogen. We found that pretreatment with E2, but not co-treatment or post-treatment, inhibited LPS-induced microglial activation, which indicates that the action of E2 is relatively upstream. Once the signaling cascades pass certain levels, E2 does not stop the already activated signals. When microglia are activated, large Kir currents are expressed within 12 h. The inward rectifying current thus can be considered an early microglial activation marker^40^. The Kir channels maintain microglial membrane potential at a hyperpolarized level, which is critical for the influx of Ca^2+^ and the subsequent production of superoxide anions^24^,^30^. Blocking Kir activity using Kir-specific inhibitor significantly suppressed the Ca^2+^ influx and ATP-induced superoxide production in microglia^41^,^42^. We found that E2 inhibited Kir, i.e., decreased I_K(IR)_ by reducing the probability that the channel would open. The inhibitory effect of E2 on the Kir2.1 channel occurred within seconds, which suggested that E2 affects the function rather than the expression levels of the Kir2.1 channel. Whether estrogen directly binds to Kir channels and hence inhibits the opening of Kir channels or indirectly modulates the function of Kir channels via post-translational modifications is an interesting research topic for future studies. Moreover, the concentrations of E2 that we used to inhibit Kir2.1 channel are much higher than the physiological range of E2 in the brain (0.08–0.19 ng/g wet weight)^43^. Considering the narrow intercellular space (e.g., the synaptic cleft) in the brain parenchyma, the local concentration of estrogen in the interstitial fluid could be several orders of magnitude higher than the concentration in brain homogenate. Micromolar concentrations of the E2 have frequently been used to demonstrate the neuroprotective effect of E2 in cells that underwent cytotoxic or hypoxic agent treatments^44^. However, high concentrations of steroid might affect membrane fluidity and subsequently the probability of channel opening. Incubating endothelial cells with millimolar concentrations of cholesterol has been shown to decrease the amplitude of inwardly rectifying K^+^ current^45^. Therefore, the effect of E2 on Kir2.1 channel opening requires further validation. Nonetheless, our findings suggest that a potential mechanism for estrogen’s regulation of microglial activation might be by controlling membrane potential through Kir2.1.

We cannot exclude the possibility that other mechanisms are also involved in the E2-induced anti-microglial activation *in vivo*. Estrogen and estrogen receptor complexes activate CCAAT/enhancer-binding proteins (C/EBPs), transrepression molecules, which then inhibit the production of proinflammatory cytokines in BV2 microglial cells^26^. Estrogen inhibits inflammatory gene expression by blocking NF-кB nuclear translocation, inhibiting NF-κB DNA binding activity, and upregulating IκB, an inhibitor of NF-κB signaling in macrophages^39^,^46^. Furthermore, estrogen and estrogen receptor complexes downregulate TLR4 expression and inhibit proinflammatory cytokine expression in macrophages^47^. It has been suggested^48^ that estrogen receptor α isotype is critical for estrogen-induced anti-inflammation, including the suppression of inflammation-related signaling pathways.

In addition to morphological and protein expression changes, cell proliferation is one of the responses of microglial activation^22^,^49^. We found that the number of microglia increased with age and LPS treatment. These increases were less dramatic in female than in male mice. Furthermore, the age-associated proliferation of microglia was upregulated by the OVX. Treatment with E2 supplements, however, counteracted the OVX-induced proliferation, all of which suggested that estrogen inhibits microglial activation-associated proliferation. Interestingly, a cell-cycle regulatory role of K^+^ channels was proposed a quarter of a century ago^50^,^51^. Treating primary cultured microglia with Kir blocker inhibited their proliferation^50^. Therefore, the estrogen-induced inhibition of age-associated microglial proliferation might also use the mechanism of Kir inhibition. Thus, an estrogen deficiency might increase cell proliferation, which might explain why the number of microglia is higher in older male mice and in female mice with an OVX.

This study was not designed to examine the direct neuroprotective effect of estrogen. There are at least three known mechanisms for the neuroprotective actions of estrogen. First, estrogen increases neuron survival by increasing anti-apoptotic genes and pro-survival PI3K-Akt signaling^16^. Second, estrogen elevates anti-oxidative capacity in neurons by increasing mitochondria glutathione levels^52^. Third, estrogen increases mitochondrial bioenergetics by upregulating the activities of electron transport chain complexes I and II^53^,^54^. In addition to these mechanisms, we showed that estrogen inhibited age-related and LPS-induced microglial activation. Because microglial activation is intimately associated with the survival of DA neurons^2^,^4^,^6^, the anti-inflammatory effect of estrogen can be considered an alternative neuroprotective mechanism, especially in the SN.

The risk that women will develop PD increases sharply after menopause^55^,^56^. To mimic the estrogen supplements given to postmenopausal women, female mice underwent an OVX and were not treated with E2 supplements until two months post-surgery. We found that age-associated microglial activation in the SN increased 3 months after an OVX. Others^57^ have reported that proinflammatory cytokine levels in the peripheral circulation were higher than normal after menopause. We also found that 1 month of E2 supplements significantly decreased OVX-induced microglial activation, which indicated that E2 is capable of preventing further activation of microglia, hence holding the microglia in a younger and less active status. However, 1 month of E2 supplements did not affect OVX-induced DA neuron losses. Apparently, E2 supplements begun 2 months post-OVX cannot revive the DA neurons already lost. This argument is supported by our observations that DA neuron loss and microglial activation were completely blocked in mice treated with E2 supplements immediately after the OVX. This suggests that estrogen replacement therapy might provide maximal neuronal protection when it is given near the onset of menopause or immediately after an OVX.

In conclusion, we characterized the temporal profiles of microglial activation and DA neuron loss in male and female mice. Age-related and LPS-induced microglial activation and DA neuron loss were more prominent in male mice, which is associated with the ovarian hormone estrogen. We found that estrogen inhibited microglial activation by controlling the membrane potential through Kir2.1. Because microglial activation is a common component of neurodegenerative diseases, clarifying that estrogen protects against microglial activation might benefit patients with PD and other neurodegenerative disorders.

## Materials and Methods

### Animals

All experiments were performed in accordance with the National Institutes of Health Guideline for animal research (Guide for the Care and Use of Laboratory Animals) and approved by the National Cheng Kung University Institutional Animal Care and Use Committee (IACUC number 101065). Male and female C57BL/6J mice (3, 6, 9, and 12 months old) and BALB/c mice (3, 6, and 9 months old) were obtained from National Cheng Kung University’s Laboratory Animal Center. Mice were housed (five per cage) with a stable temperature (24 ± 1 °C), a 12-h light/dark cycle, and unrestricted access to food and water. Clean solid-bottom cages with bedding were changed weekly. The body weight of C57BL/6J mice increased with age (Table S1). The housing environment (every month) and animal health (every 3 months) were monitored by the Laboratory Animal Center. An example of the Health monitoring annual report (from Jan 24, 2015, to Jan 25, 2016) is shown in the Table S2.

### LPS dosing treatment

At 3 months old, the mice were subdivided into LPS and Saline (control) groups. The LPS group was intraperitoneally (i.p.) injected with a single dose (0.05, 0.1, or 0.15 mg/kg) of LPS, and the Saline group was injected (i.p.) with an equivalent volume of saline. LPS, a major constituent of the outer membrane of Gram-negative bacteria, is a heterogeneous group of molecules that triggers innate immune responses through TLR4. We used the LPS strain 055: B5 (Sigma-Aldrich, St. Louis, MO), which induces strong peripheral inflammatory responses and microglial activation^35^.

### Ovariectomy procedure and 17β-estradiol supplement

Two-month-old female mice were anesthetized with chloral hydrate (400 mg/kg) (Sigma-Aldrich) and randomly divided into Ovariectomy (OVX) and Sham groups. The OVX group underwent a bilateral OVX, and the Sham group was given the same surgical incisions, but their ovaries were not removed. The mice were placed in clean cages, kept warm, and observed until they had recovered from anesthesia. One month later, they were injected (i.p.) with 0.15 mg/kg of LPS and killed 24 h later.

In another study, 6-month-old mice were given an OVX. Some were given E2 supplements for the immediately following 3 months, and some for only 1 month (from 8 to 9 months old). The E2 solution contained 36 μg of E2 (E8875; Sigma-Aldrich) dissolved in 1 ml of propylene-glycol (Sigma-Aldrich) and filled in the Alzet osmotic mini-pump (Model 1004 [100 μl]; Durect, Cupertino, CA) at a rate of 0.11 μl/h for 28 days. The mini-pump was implanted subcutaneously, superior to the scapular. For the 3-month E2 supplement group, the osmotic mini-pump was replaced with a refilled mini-pump once per month. Mice implanted with propylene-glycol-filled osmotic mini-pumps were Vehicle controls.

### 17β-estradiol and LPS treatment

Three-month-old male mice were given intraperitoneal or intracranial injection of E2 before LPS injection. In the intraperitoneal injection study, E2 (0, 10 and 100 μg/kg) was given 1 hour before the mice were intraperitoneally injected with 0.15 mg/kg of LPS. The mice were given an overdose of chloral hydrate one day after the LPS injection. In the intracranial injection study, 1 μl of E2 (100 pg/ml, 0.1% DMSO) was injected into the right SN (stereotaxic coordinates in mm from the bregma: anterior/posterior, −0.29; lateral, + 0.12; ventral, −0.42). An equal volume of saline was injected into the left SN to create an internal sham control. The infusion was controlled using a syringe pump at a rate of 0.1 μl/min. The needle was removed 20 min after the infusion was completed. One hour later, the mice were intraperitoneally injected with 0.15 mg/kg of LPS and given an overdose of chloral hydrate one day after the LPS injection.

### Preparing tissue

Brain tissue was prepared as previously described^33^. Briefly, mice were anesthetized with an overdose of chloral hydrate and perfused from the left ventricle with ice-cold PBS. Their brains were removed, post-fixed in 4% paraformaldehyde for 48 h at 4 °C, and cryoprotected with a 30% sucrose solution. The tissue was then coronally sliced into 30-μm sections using a freezing microtome, and stored in cryoprotectant at −20 °C until it was used.

### Immunohistochemistry

The protocol for immunohistochemical staining has been described elsewhere^35^. The 4% paraformaldehyde-fixed brain sections were stained using rabbit anti-ionized calcium-binding adapter molecule-1 (Iba1) (1:2000) (Wako Pure Chemical Industries, Osaka, Japan) and rat anti-CD11b (1:200) (R&D Systems, Minneapolis, MN) for microglia, mouse anti-major histocompatibility complex (MHC) class II (1.:250) (AbD Serotec, Oxford, UK) for activated microglia and rabbit anti-tyrosine hydroxylase (TH) (1:2000) (Chemicon, Temecula, CA) for DA neurons. Brain specimens were incubated with appropriate biotin-conjugated secondary antibodies and avidin-biotin peroxidase (ABC; Vector Laboratories, Burlingame, CA) using diaminobenzidine as the substrate. The signals were evaluated by the morphologies and intensities after subtracting the signals of the primary antibody-omitted negative controls. In some cases, the primary antibodies were replaced by isotype antibodies (normal anti-rabbit, anti-rat and anti-mouse antibodies) to control for non-specific binding of the primary antibodies. Incubations without primary antibody were used as negative controls.

### Dual immunofluorescence staining and confocal microscopy

Adjacent 10-μm coronal sections were incubated for 1 h in a PBS solution containing 0.1% Triton X-100 and 3% bovine serum albumin at room temperature and then transferred to a solution that contained Iba1 (1:1000) (Wako) antibodies overnight at 4 °C. Appropriate secondary antibodies, conjugated with fluorescent dye Alexa-fluor 488 (1:1000) (Invitrogen, Carlsbad, CA) were used to detect the expression of Iba1. Incubations without primary antibody were used as negative controls. Images were acquired using a confocal microscope (FV1000MPE; Olympus, Tokyo, Japan) connected to a computer equipped with imaging software (FV10-ASW; Olympus).

### Counting cells

The number of Iba1-, CD11b-, MHC class II- and TH-positive^+^ cells was counted in the right SN using a modified stereological procedure previously described^35^,^49^. Most of the TH^+^ neurons in the central nervous system are in the SN and the ventral tegmental area (VTA) of the ventral midbrain. We defined these two regions based on the following criteria: 1) The accessory optic tract separates the superomedially situated VTA from the inferolaterally located SN; 2) in regions where the accessory optic tract is not readily seen, the hypothetical line connecting the most inferior point on the dorsal curvature and the most superior point on the ventral curvature is the boundary between the SN and VTA; and 3) the somas of DA neurons in the SN are larger than those in the VTA^58^. The neurites of DA neurons in the SN project inferiorly toward the pars reticular, and the neurites of DA neurons in the VTA project superiorly.

The entire SN was cut into an average of thirty-six 30-μm coronal sections. The number of Iba1^+^, CD11b^+^, MHC class II^+^ and TH^+^ cells was counted in every 6th section. Positive cells were identified using a microscope (Carl Zeiss, Oberkochen, Germany) with a 40× objective. The number of labeled cells per section was divided by the slide selection ratio (6/36) to obtain the total number of cells in each SN. Photomicrographs were taken using a digital camera (Axiocam MRc; Carl Zeiss) connected to a computer equipped with imaging software (Axiovision 4.8; Carl Zeiss). The Iba1^+^, CD11b^+^ and MHC class II^+^ areas were obtained using image analysis software (Image-Pro Plus 6.0; Media Cybernetics, Rockville, MD) by measuring the area with Iba1^+^, CD11b^+^ and MHC class II^+^ intensity higher than a given background threshold. The background intensity threshold was fixed and used for all sections.

### Cell culture

Immortalized murine microglial BV2 cells were used to characterize the anti-inflammatory effect of E2. The BV2 cells were cultured in Dulbecco’s modified Eagle’s medium/F12, which contained 10% fetal bovine serum at 37 °C in an atmosphere containing 5% CO_2_. BV2 cells (10^6^) were seeded onto a 10-cm dish, left overnight, and then used for experiments. To antagonize LPS-induced responses, BV2 cells were treated with various concentrations of E2 (0, 10, and 100 pg/ml) before (pre-treatment), combined with (co-treatment), and after (post-treatment) adding LPS (10 ng/ml) to the culture media. In the pre-treatment group, BV2 cells were treated with E2 for 30 min, washed, and then cultured for 30 min in fresh medium containing LPS. In the co-treatment group, BV2 cells were treated with E2 and LPS together for 30 min. In the post-treatment group, BV2 cells were treated with LPS for 30 min, washed, and then cultured for 30 min in fresh medium containing E2.

### ELISA for TNF-α quantification

A mouse ELISA kit (TNF-α Antibody Pair; Invitrogen) was used to quantify the levels of TNF-α in the medium after LPS and E2 treatment. The plate was read in an ELISA-spectrophotometer reader with an absorbance wavelength of 405 nm. Standard curves were obtained from values generated from known concentrations of TNF-α provided by the kits.

### Western blotting

SN tissues and BV2 cells were homogenized (1:1, weight:volume in SN; 1:3, in cells) in RIPA buffer (1% NP40, 1 mM of phenylmethanesulfonyl fluoride, 10 μg/ml of aprotinin, 1 μg/ml of leupeptin, 0.5 mM of sodium vanadate, 137 mM of NaCl, 20 mM of Tris-HCl [pH 8.0]) containing 0.5 M dithiothreitol and a mixture of protease inhibitors (Mini Protease Inhibitor Cocktail Tablets; Roche Diagnostics, Mannheim, Germany) and then centrifuged at 13,000 × g at 4 °C for 30 min. The supernatants were collected and protein concentrations were determined. The supernatants (30 μg of total protein) were heated to 70 °C for 10 min and loaded into each well of 4–12% polyacrylamide gel and resolved at 120 V for 120 min. The separated proteins were transferred to a PVDF membrane (Bio-Rad Laboratories, Hercules, CA), blocked in 5% non-fat milk, and probed with primary antibodies: TLR4 (1:20,000) (Cell Signaling, Danvers, MA), total (1:20,000) (Cell Signaling) and phosphorylation form of p38 (1:10,000) (Cell Signaling) and TNF-α (Abcam, Cambridge, UK). The bound antibodies were detected using an enhanced chemiluminescence detection kit (PerkinElmer, Boston, MA). The band densities were measured using an imaging system (BioChemi; UVP, Upland, CA) and analyzed using ImageJ (1.43u) (http://rsb.info.nih.gov/ij/). For gel loading control, membranes were reprobed with a monoclonal β-actin antibody (1:40,000) (Chemicon).

### Electrophysiological measurements

BV2 cells were harvested with 1% trypsin/EDTA solution before the experiments. An aliquot of cell suspension was transferred to a recording chamber that was mounted on the mechanical stage of an inverted fluorescent microscope (CKX-41; Olympus, Tokyo, Japan) coupled to a digital video system (DCR-TRV30; Sony, Tokyo, Japan) with a magnification of ~1500x. The cells were immersed at room temperature (20–25 °C) in normal Tyrode’s solution that contained 1.8 mM of CaCl_2_, 136.5 mM of NaCl, 5.4 mM of KVl, 1.8 mM of CaCl_2_, 0.53 mM of MgCl_2_, 5.5 mM of glucose, and 5.5 mM of HEPES-NaOH buffer (pH 7.4). In single-channel current recordings, the pipette solution contained 145 mM of KCl, 2 mM of MgCl_2_, and 5 mM of HEPES-KOH buffer (pH 7.2). The electrodes were pulled from Kimax-51 capillaries (#34500; Kimble Glass, Vineland, NJ) in a micropipette puller (P-97 Flaming/Brown; Sutter, Novato, CA), and their tips were fire-polished using a microforge (MF-83; Narishige Scientific Instrument Lab., Tokyo, Japan). These electrodes, which had a resistance of 3–5 MΩ when filled with different intracellular solutions, were mounted on and controlled by a hydraulic micromanipulator (WR-98; Narishige). Ion currents recorded in whole-cell mode were measured using a standard patch-clamp technique with a patch-clamp amplifier (Axopatch 200B; Molecular Devices, Sunnyvale, CA)^29^. Junctional potentials between the internal pipette solution and extracellular medium were nulled before seal formation.

The data were stored online in a TravelMate-6253 laptop computer (Acer, Taipei, Taiwan) at 10 kHz through a Digidata-1440A interface (Molecular Devices) which was controlled by pCLAMP 10.2 software (Molecular Devices)^29^. Current signals were low-pass filtered at 3 kHz. The signals were stored and subsequently analyzed using different tools: pCLAMP 10.2, LabChart 7.0 (AD Instruments; Gerin, Tainan, Taiwan), Origin 8.0 (OriginLab, Northampton, MA), and custom-made macro procedures built using Excel 2010 in Windows 7 (Microsoft, Redmond, WA). The voltage-step profiles with either rectangular or ramp pulses created from pCLAMP 10.2 were commonly employed to determine the current-voltage (*I-V*) relations for the ion currents (e.g., I_K(IR)_).

### Statistical Analysis

Data are mean ± SEM. Two-way analysis of variance (ANOVA) was used to analyze the two main effects (gender/age, gender/LPS, or OVX/LPS) and possible interactions between them on the area and number of Iba1^+^, CD11b^+^, MHC class II^+^ and TH^+^ cells. Bonferroni post-hoc tests were done if the main effects or interactions were significant (*p* < 0.05). The effect of LPS on TNF-α production and the effect of estrogen on LPS-induced microglial activation were analyzed using one-way ANOVA and then Bonferroni post-hoc tests if the overall effect was significant (*p* < 0.05). Paired two-tailed Student’s t tests were used to analyze the directly effect of intra-nigral estrogen treatment on LPS-induced microglial activation.

## Additional Information

**How to cite this article**: Wu, S.-Y. *et al.* Estrogen ameliorates microglial activation by inhibiting the Kir2.1 inward-rectifier K^+^ channel. *Sci. Rep.* **6**, 22864; doi: 10.1038/srep22864 (2016).

## Supplementary Material

Supplementary Information
