# Supplementary material for: Estrogen ameliorates microglial activation by inhibiting the Kir2.1 inward-rectifier K+ channel
Source: Sci Rep. 2016 Mar 10;6:22864. doi: 10.1038/srep22864 (PMC4785403; doi:10.1038/srep22864)
Supplement: Supplementary Information [file srep22864-s1.pdf]

Supplementary materials

Estrogen ameliorates microglial activation by inhibiting the Kir2.1 inward-rectifier K^+ channel

Shih-Ying Wu*[1], Yun-Wen Chen*[2,3], Sheng-Feng Tsai[1], Sheng-Nan Wu[4], Yao-Hsiang Shih[1], Ya-Fen Jiang-Shieh[2], Ting-Ting Yang[3,#], Yu-Min Kuo[1,2,#]

[1]Institute of Basic Medical Sciences, National Cheng Kung University, Tainan, Taiwan

[2]Department of Cell Biology and Anatomy, National Cheng Kung University, Tainan, Taiwan

[3]Department of Chinese Medicine for Post-Baccalaureate, I-Shou University, Kaohsiung, Taiwan

[4]Department of Physiology, National Cheng Kung University, Tainan, Taiwan

*These authors contributed equally to this paper.

#Corresponding authors:

T. T. Yang, Department of Chinese Medicine for Post-Baccalaureate, I-Shou University, Kaohsiung, Taiwan. E-mail: tingting.taiwan@gmail.com

Y. M. Kuo, Department of Cell Biology and Anatomy, National Cheng Kung University Medical College. 1 Ta Hsueh Road, Tainan 70101, Taiwan. Tel.: +886-6-235-3535 ext. 5294; Fax: +886-6-209-3007. E-mail: kuoym@mail.ncku.edu.tw

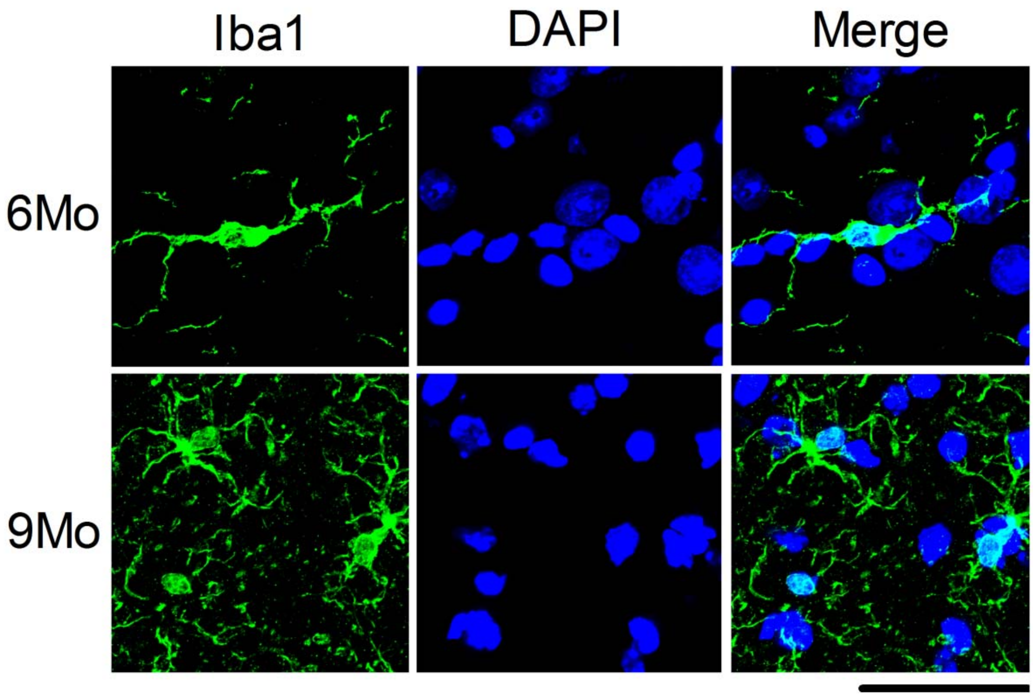

Supplementary Figure S1. Confocal immunofluorescence microscopy images of Iba1⁺ cells in the SNs of 6- and 9-month-old male mice. Scale bar: 20 µm. 6Mo: 6-month-old; 9Mo: 9-month-old.

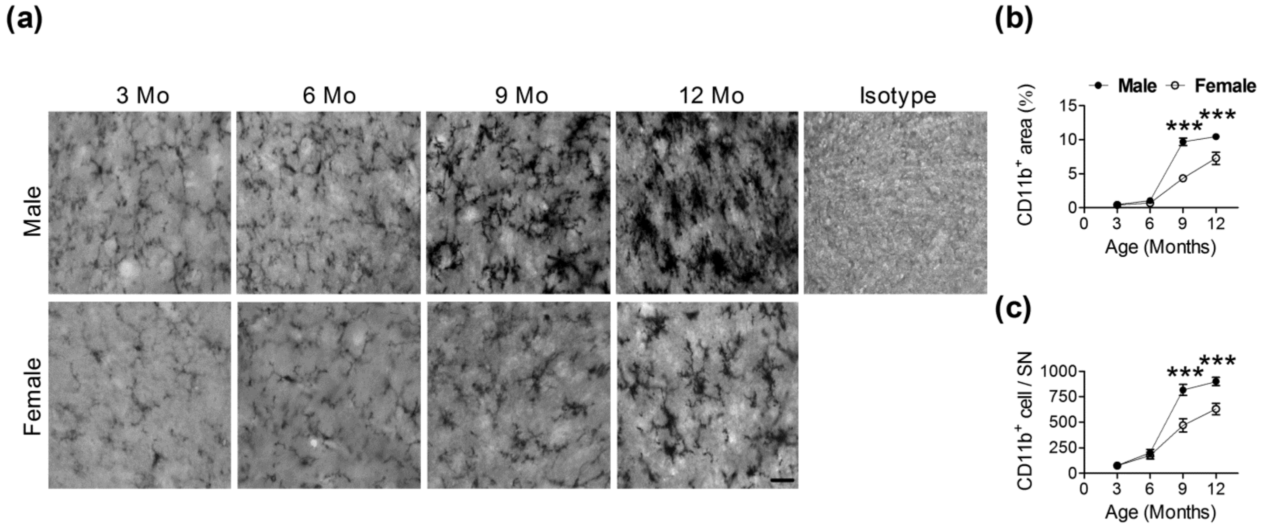

Supplementary Figure S2. Temporal profiles of microglia in the SN of female and male C57BL/6J mice. (a) Representative immunostained micrographs show CD11b$^+$ cells in the SN of 3-, 6-, 9-, and 12-month-old female and male mice. Scale bar: 10 µm. Quantitative results of CD11b$^+$ cell areas (**b**) and the number of Iba1+ cells (**c**) in the SN of female and male mice at different ages (n = 5). *** ($p < 0.001$): versus the opposite sex.

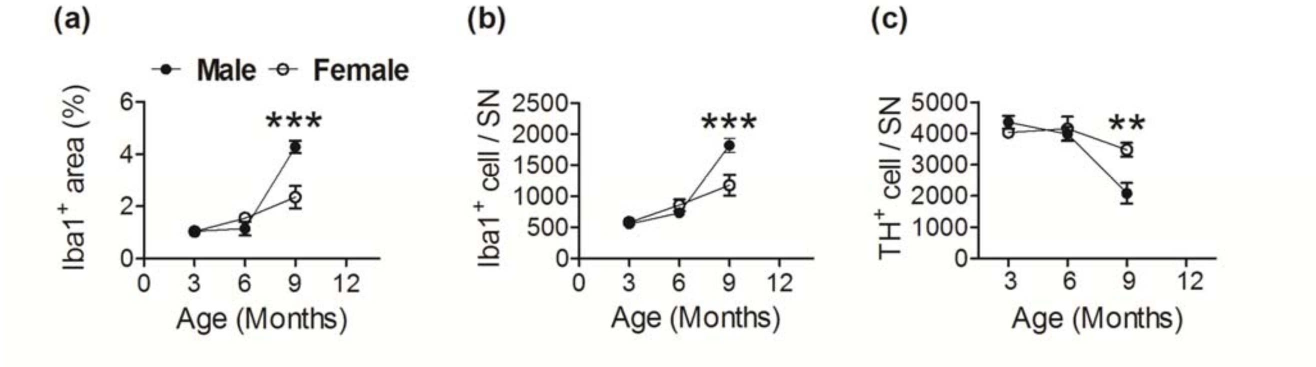

Supplementary Figure S3. Temporal profiles of microglia and DA neurons in the SN of female and male BALB/c mice. (**a,b,c**) Quantitative results of Iba1$^+$ cell areas (**a**) and the number of Iba1$^+$ cells (**b**) and the number of TH$^+$ cells (**c**) in the SN of female and male mice at different ages (n = 4). ** ($p < 0.01$), *** ($p < 0.001$): versus the opposite sex.

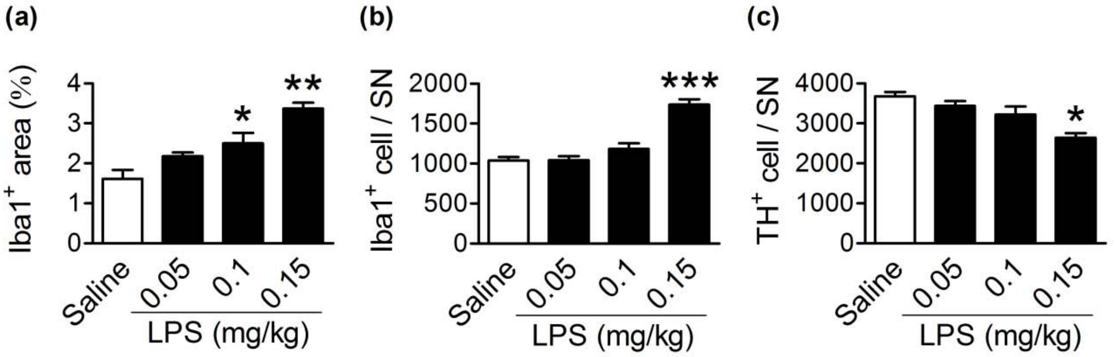

Supplementary Figure S4. Dose effects of LPS on microglial activation and DA neuron loss in the SN of 3-month-old male mice. Dose effects of LPS on (**a**) Iba1$^+$ cell areas, (**b**) the number of Iba1$^+$ cells, and (**c**) the number of TH$^+$ cells in the SN of 3-month-old male mice (n = 4). * ($p < 0.05$), * * ($p < 0.01$), *** ($p < 0.001$): versus Saline controls.

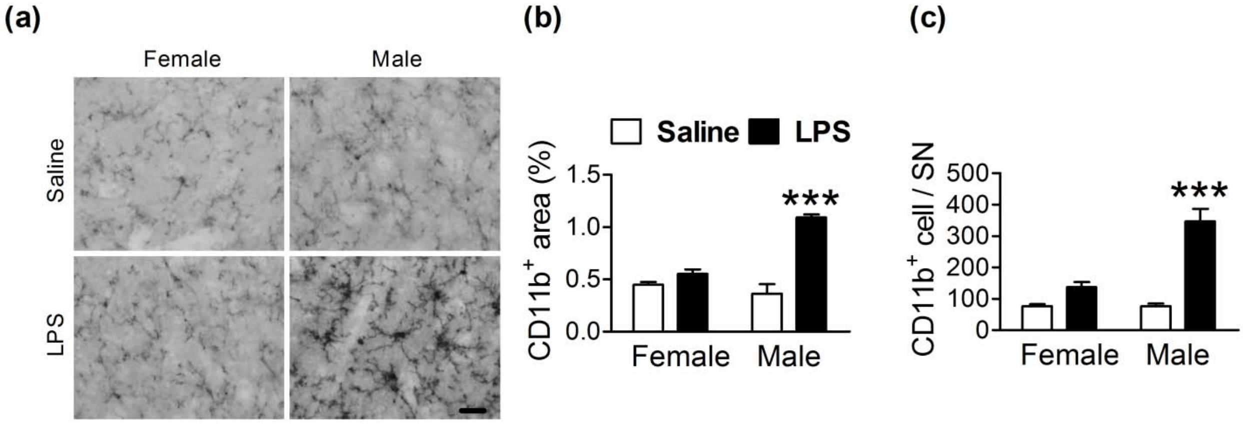

Supplementary Figure S5. Differential effects of LPS on microglial activation in the SN of female and male mice. (**a**) Representative immunostained micrographs show $CD11b^{+}$ cells in the SN of female and male mice 1 day after an LPS (0.15 mg/kg of body weight, i.p.) injection. Scale bar: 10 µm. Quantitative results of $CD11b^{+}$ cell areas (**b**) and the number of $CD11b^{+}$ cells (**c**) in the SN of female and male mice 1 day after an LPS injection (n = 5-6). *** ($p < 0.001$): versus respective Saline group.

Supplementary Table S1. Body weight of C57BL/6J mice.

Age (months)		Sample size	Mean ± SEM (g)	coefficient of variation (%)
3	male	8	21.7 ± 0.7	9.2
	female	10	21.4 ± 0.3	4.8
6	male	8	26.2 ± 0.6	6.2
	female	7	25.5 ± 0.8	8.4
9	male	8	29.7 ± 0.6	5.6
	female	5	28.2 ± 0.6	4.9
12	male	4	33.3 ± 1.1	6.6
	female	7	29.6 ± 0.8	7.5

Supplementary Table S2. The health monitoring annual report of C57BL/6J mice.

Laboratory Animal Center of National Cheng Kung University

Health monitoring report

Location：Tainan, Taiwan　　　Report date：25 Jan,2016

Area：12F Clean　　　ROOM：CH32-H　　　Specics：Mouse

	Current POS/TSTD	12Months POS/TSTD	Methods
Mouse Hepatitis virus	0/1	0/5	ELISA
Pneumonia Virus of Mice	0/1	0/5	ELISA
TMEV	0/1	0/5	ELISA
Mouse adenovirus (MAd)	0/1	0/5	ELISA
Mouse parvovirus (MPV)	0/1	0/5	ELISA
Mycoplasma pulmonis	0/1	0/5	ELISA
Murine norovirus	0/1	0/5	ELISA
Minute virus of mice (MVM)	0/1	0/5	ELISA
Hantaan virus	0/1	0/2	ELISA
Sendai virus	0/1	0/3	ELISA
Ectromelia virus (Mouse Pox)	0/1	0/3	ELISA
Reovirus 3 (Reo 3)	0/1	0/3	ELISA
LCMV	0/1	0/2	ELISA
Bordetella bronchiseptica	0/1	0/3	Culture
Corynebacterium kutscheri	0/1	0/3	Culture
Salmonella spp.	0/1	0/3	Culture
Pseudomonas aeruginosa	0/1	0/3	Culture
Helicobacter spp.	0/1	0/3	PCR
Pneumocystis murina	0/1	0/3	PCR
Endoparasites- Helminths			
Aspiculuris tetraptera	0/1	0/3	Direct exam. and flotation test
Hymenolpis diminuta	0/1	0/3	Direct exam. and flotation test
Rodentolepis nana	0/1	0/3	Direct exam. and flotation test
Syphacia spp.	0/1	0/3	Direct exam. and flotation test
Ectoparasites			
Flea	0/1	0/3	Clinical exam and necropsy
Louse	0/1	0/3	Clinical exam and necropsy
Mite	0/1	0/3	Clinical exam and necropsy

國立成功大學 實驗動物中心 醫學院
